# Evaluation of Multiplex-Based Antibody Testing for Use in Large-Scale Surveillance for Yaws: a Comparative Study

**DOI:** 10.1128/JCM.02572-15

**Published:** 2016-04-25

**Authors:** Gretchen M. Cooley, Oriol Mitja, Brook Goodhew, Allan Pillay, Patrick J. Lammie, Arnold Castro, Penias Moses, Cheng Chen, Tun Ye, Ronald Ballard, Diana L. Martin

**Affiliations:** a Centers for Disease Control and Prevention, Atlanta, Georgia, USA; b Lihir Medical Center, International SOS, Newcrest Mining, Lihir Island, Papua New Guinea; c University of Barcelona, Barcelona, Spain

## Abstract

WHO has targeted yaws for global eradication by 2020. The program goals are to interrupt the transmission in countries where yaws is endemic and to certify countries as yaws free where yaws was endemic in the past. No new rapid plasmin reagin (RPR) seroreactivity in young children is required for certification of elimination at a country level. We sought to evaluate whether antibody responses to specific treponemal antigens measured in a high-throughput multiplex bead array (MBA) assay differentiate past versus current infection and whether a nontreponemal lipoidal antigen test can be incorporated into the MBA. Serum and dried blood spot specimens collected for yaws surveillance projects in Ghana, Vanuatu, and Papua New Guinea (PNG) were run on MBA to measure antibodies against recombinant p17 (rp17) and treponemal membrane protein A (TmpA) treponemal antigens. Results were compared to standard treponemal laboratory (TPPA or TPHA [TPP(H)A]) and quantitative RPR test data. Of 589 specimens, 241 were TPP(H)A^+^/RPR^+^, 88 were TPP(H)A^+^/RPR^−^, 6 were TPP(H)A^−^/RPR^+^, and 254 were negative for both tests. Compared to TPP(H)A, reactive concordance of rp17 was 93.7%, while reactive concordance of TmpA was only 81.9%. TmpA-specific reactivity showed good correlation with RPR titers (*R*^2^ = 0.41; *P* < 0.0001). IgG responses to the lipoidal antigen used in RPR testing (cardiolipin) were not detected in the MBA. Our results suggest that TmpA can be used as a treponemal antigen marker for recent or active infection and potentially replace RPR in a high-throughput multiplex tool for large-scale yaws surveillance.

## INTRODUCTION

Yaws is an infectious disease caused by Treponema pallidum ssp. *pertenue*, which mainly affects children in rural communities in the humid tropics (1, 2). This bacterium causes a chronic relapsing disease characterized by highly contagious primary and secondary cutaneous lesions and noncontagious tertiary destructive lesions of the bones. It is spread by skin-to-skin contact and is not sexually transmitted but cannot be distinguished serologically from syphilis (3).

Clinical features alone are not reliable indicators of yaws infection, with recent surveys indicating that only 30% to 60% of all suspected skin ulcers were seroreactive using conventional serological tests for syphilis (4, 5). Therefore, diagnosis of yaws typically requires confirmatory serological testing. Typically, a serological diagnosis for yaws is based on the detection of two distinct antibodies: one against a treponemal antigen that indicates current or previous exposure to infection, such as the Treponema pallidum particle agglutination (TPPA) and hemagglutination (TPHA) assays, and one against cardiolipin, a nontreponemal antigen that can be quantitatively measured by tests, such as the rapid plasma reagin (RPR) and Venereal Disease Research Laboratory (VDRL) tests, that are better able to discriminate between current active disease and past infections.

WHO has targeted yaws eradication by 2020 and has developed the Morges strategy, which is comprised of mapping the disease at a community level and subsequently treating the entire population of communities in which yaws is endemic with single-dose azithromycin (5, 6). The efficacy of this approach was demonstrated in a study of mass treatment in Papua New Guinea (PNG) (5) and in Vanuatu and a subdistrict of southern Ghana (A. Pillay, C. Chen, and T. Ye, unpublished data). The program goals are to interrupt transmission in all countries where the disease is currently endemic and to certify that all previously countries where the disease was endemic are yaws free. Certification of elimination at a country level is determined by zero reporting of yaws cases and by lack of RPR seroreactivity in young children, because reactivity of nontreponemal tests is a better indicator of recent infection than reactivity of treponemal tests. Unfortunately, the RPR test cannot be automated for use in large-scale serosurveys.

A large-scale serosurveillance tool for use in stand-alone surveys or in conjunction with serosurveillance for other neglected tropical diseases (NTDs) is required to direct mapping efforts and to certify elimination of yaws (7–10). Ideally, this tool would be able to differentiate previous from current treponemal infection. We therefore sought to evaluate serological testing for yaws by use of two recombinant treponemal antigens, the recombinant p17 (rp17) antigen and the treponemal membrane protein A (TmpA) on a multiplex bead array (MBA) assay, which is capable of measuring multiple analytes in a single run. These data were compared to reference standards and correlated with results obtained quantitatively against the RPR test. Some evidence suggests that antibody titers to certain treponemal antigens, primarily TmpA, decline rapidly following treatment for syphilis (11, 12). TmpA may therefore be a potential candidate antigen to measure the impact of yaws elimination efforts. A secondary aim of our study was to evaluate the performance of the nontreponemal antigen cardiolipin when linked to beads on the MBA assay.

## MATERIALS AND METHODS

### Study population and ethics statement.

Specimens were collected as part of various yaws evaluation studies in Ghana, Vanuatu, and PNG in 2013. In Ghana, serum samples were collected from children aged 5 to 14 years as part of a baseline assessment of the prevalence of yaws. In Vanuatu, whole blood was collected from children aged 5 to 14 years onto filter paper (TropBio Pty Ltd., Queensland, Australia) and then allowed to air dry at least 4 h to form dried blood spots (DBS). DBS were collected from children with clinically suspected yawslike lesions as part of the baseline assessment of the prevalence of yaws in schoolchildren from randomly selected primary schools. In PNG, serum samples were collected from children aged 1 to 14 years during serological surveys for latent yaws, as described elsewhere (5). The studies were reviewed and approved by the Ghana Health Service Ethical Review Committee for Ghana; by the Ministry of Health in Vanuatu, WHO, and the CDC for Vanuatu; and by the Medical Research Advisory Committee of the Papua New Guinea Ministry of Health for PNG. All identifiers were removed prior to shipment to CDC for serological testing, and CDC staff members were determined to be nonengaged in human subject research.

### Serological testing.

Blood specimens were tested for seroreactivity using the TPPA test (used in Ghana and Vanuatu; Fujirebio Diagnostics, Malvern, PA,), the TPHA test (used in PNG; Human Diagnostics, Wiesbaden, Germany) (for brevity, we refer to the treponemal testing as TPP[H]A), and a quantitative RPR test (Alere Wampole, Waltham, MA, for Ghana and Vanuatu; Human Diagnostics, Wiesbaden, Germany, for PNG). All assays were performed according to the manufacturers' instructions. RPR data were recorded as titers: RPR negative, R1 (positive using neat serum), R2 (positive at 1:2 dilution), R4 (1:4), R8 (1:8), R16 (1:16), R32 (1:32), R64 (1:64), or R128 (>1:128).

### Antigen bead coupling.

### Proteins.

Recombinant glutathione *S*-transferase (GST)-tagged rp17 (Chembio Diagnostic Systems, Inc., Medford, NY) and full-length recombinant TmpA (ViroGen, Watertown, MA) were provided as a 114-kDa fusion at the N terminus with β-galactosidase. Both rp17 and TmpA were dialyzed overnight at 4°C into phosphate-buffered saline (PBS). Antigens were coupled to polystyrene microspheres (SeroMap beads; Luminex Corporation, Austin, TX) as previously described (1). Briefly, carboxyl groups on the beads were chemically modified to ester groups by 1-ethyl-3(3-dimethlaminopropyl) carbodiimide (EDC) (Calbiochem, San Diego, CA) in the presence of *N*-hydroxysulfosuccinimide (NHS) (Pierce, Rockford, IL). Primary amine groups on the antigens were then reacted with ester groups on the beads to create amide covalent bonds. Antigen-coupled beads were quantified by hemocytometer and stored at 4°C with PBS containing 1% bovine serum albumin (BSA) plus protease inhibitors.

### Cardiolipin.

Poly-l-lysine (Sigma-Aldrich Co. LLC., St. Louis, MO) and cadavarine (Sigma-Aldrich) were tested as diamine linkers to conjugate the carboxylated cardiolipin (Avanti Polar Lipids, Inc., Alabaster, AL) to the carboxylated polystyrene beads. Poly-l-lysine and cadavarine were serially diluted in 0.1 M NaH_2_PO_4_ (pH 5.6) (NaP) to four different concentrations (1,000, 250, 62.5, and 15.6 μg/ml) for a total of eight amination conditions. Polystyrene beads (106) were pelleted and washed two times with NaP and activated in 200 μl NaP plus 60 mg/ml EDC and 20 mg/ml NHS. Beads were incubated for 30 min at room temperature (RT) with EDC and NHS, and then 100 μl of each amination condition was added and incubated for 1 h at RT with shaking. Beads were then blocked with PBS plus 1% BSA, washed with PBS, and suspended in 100 μl PBS (pH 7.2). Carboxylated cardiolipin was activated by incubation with 200 μl of 60 mg/ml EDC and 100 μl of 20 mg/ml NHS for 1 h at RT. Activated cardiolipin was coupled to amine beads by incubating 100 μl of activated cardiolipin with 100 μl of amine beads for 2 h at RT with stirring. Beads were then washed and resuspended in bead storage buffer and stored at 4°C until use.

### Blood spot and serum preparation.

One blood spot extension (calibrated to hold **∼**10 μl whole blood) was eluted overnight at 4°C with 2,000 μl PBS containing 0.5% casein (Sigma C7078), 0.05% Tween 20, 0.02% sodium azide, 0.5% polyvinyl alcohol (PVA), 0.8% polyvinylpyrrolidone (PVP), and 3 μg/ml Escherichia coli crude extract containing recombinant GST (PCN-EC) for a final whole-blood dilution of 1:400 of eluted sera (assuming that 50% of DBS content is serum). Serum samples were diluted 1:400 in 500 μl PCN-EC. Dilutions were incubated at 37°C for 1 h to increase the effectiveness of the E. coli extract absorption of nonspecific antibodies. Dilutions were stored at 4°C and centrifuged at 16,000 × *g* immediately before use. For cardiolipin, serum samples were diluted in PCN-EC at 1:6, 1:25, 1:100, and 1:400.

### Multiplex bead assay.

Blood spot eluates and diluted sera were run on the Luminex 200 as previously described (1). For detection of rp17 or TmpA-specific IgG, samples were incubated with antigen-coated microspheres; then, following removal of serum, bound antibody was detected with biotinylated anti-human IgG and anti-human IgG4 antibodies and detected using streptavidin-conjugated phycoerythrin (PE). The fluorescent signal emitted by the PE was expressed as the median fluorescence intensity minus background (MFI-BG). For detection of cardiolipin-specific IgG or IgM, specimens were run separately with cardiolipin-coupled microbeads, followed by biotinylated anti-human IgG or IgM detection antibody (Southern Biotech) at 50 ng/well. Detection with streptavidin-PE was performed as described above.

### Statistical analysis.

Statistical analyses were performed using GraphPad Prism 6.0 software (GraphPad Software, Inc., La Jolla, CA). Mann-Whitney tests were used for comparison among all three countries. We used a log-normal regression model to test whether the median fluorescent intensity of MBA assays increased as a function of RPR titer. Linear regression analysis of data from three countries combined was performed using best-fit values of a log-normal regression model. *R*^2^ was tested by goodness-of-fit analysis, and differences in slopes were calculated with Fisher's exact test.

## RESULTS

### Demographic and standard serology testing.

Overall, 592 specimens were tested: 255 from Ghana, 169 from Vanuatu, and 168 from PNG. Of the 592 specimens, 241 (41.1%) had positive RPR and TPP(H)A results, 88 (14.9%) were positive for TPP(H)A alone, 6 (1.0%) had positive RPR but negative TPP(H)A results, and the remaining 254 specimens (42.9%) were nonreactive in both the treponemal and nontreponemal reference tests.

### Sensitivity and specificity of antigens.

Receiver operator curves were generated for each antigen using a panel of pediatric sera from countries in which yaws was not endemic for the negative population and sera or blood spots from children <15 years old from Ghana and Vanuatu who were TPPA positive and had RPR values of >R04 for the positive population. A cutoff that maximized combined sensitivity and specificity was set at 4,702 MFI for rp17 (100% sensitive, 100% specific) and 285 MFI for TmpA (97% sensitive, 100% specific).

The overall sensitivity of the rp17 assay compared to TPP(H)A was 90.1% (95% confidence interval [CI], 86.84 to 93.28), with a specificity of 97.6% (95% CI, 95.79 to 99.51) (Table 1). The TmpA assay showed a sensitivity of 67.47% (95% CI, 62.43 to 72.51) and a specificity of 99.2% (95% CI, 98.14 to 100) versus TPP(H)A. The overall sensitivity of the rp17 assay compared with RPR was 94.8% (95% CI, 92.02 to 97.54), while the sensitivity for TmpA compared to RPR was 79.9% (95% CI, 74.94 to 84.9). The overall specificity of rp17 compared to the RPR assay was 92.7% (95% CI, 89.59 to 95.76), and the specificity of TmpA compared to RPR was 98.2% (95% CI, 96.57 to 99.76). The country-specific sensitivities and specificities of rp17 and TmpA-specific antibody reactivity against TPP(H)A or RPR testing are shown in Table 1. Specificities were not calculated for specimens from PNG because only a convenience sample of TPP(H)A-positive specimens was provided for testing and analysis.

**TABLE 1:** Sensitivities and specificities of antibody responses against treponemal antigens rp17 and TmpA to standard serological tests^*a*^

	N	rp17 v TPP(H)A (95% CI)	TmpA v TPP(H)A (95% CI)	rp17 v RPR (95% CI)	TmpA v RPR (95% CI)
Sensitivity					
Total	587	90.1 (86.84–93.28)	67.47 (62.43–72.51)	94.8 (92.02–97.54)	79.9 (74.94–84.9)
Ghana	255	95.0 (90.73–99.27)	89.0 (82.87–96.98)	98.9 (96.67–100)	96.6 (92.88–100)
Vanuatu	169	89.9 (82.72–96.98)	50.7 (38.92–62.52)	88.7 (80.83–96.59)	56.5 (44.11–68.79)
PNG	163	87.1 (81.97–92.25)	61.3 (53.87–68.83)	94.9 (90.54–99.26)	79.6 (71.61–87.57)
Specificity					
Total	424	97.6 (95.79–99.51)	99.2 (98.14–100)	92.7 (89.59–95.76)	98.2 (96.57–99.76)
Ghana	255	96.8 (93.99–99.55)	98.7 (96.93–100)	92.8 (88.83–96.71)	97.0 (94.39–99.59)
Vanuatu	169	99.0 (97.05–100)	100.0 (100–100)	92.5 (87.54–97.5)	100.0 (94.07–100)

a Dried blood spots or serum were tested for antitreponemal antibodies using a Luminex assay and compared to field-based determination of TPP(H)A or RPR positivity. The number of specimens testing positive or negative by each assay is listed by country. 95% confidence intervals (CIs) are shown in parentheses. PNG specimens were not included in the specificity analysis because only TPHA-positive specimens were sent for testing.

### Evaluation of treponemal antigens and cardiolipin on an MBA assay.

**FIG 1:**
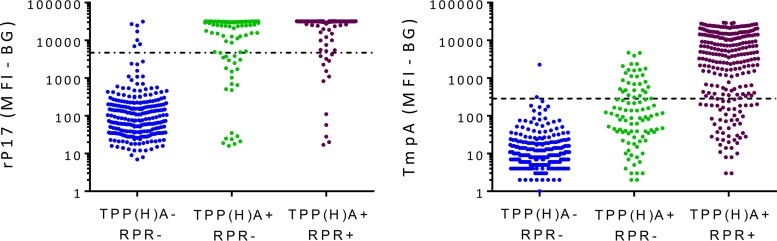
Reactivity of rp17 and TmpA by reactivity to standard diagnostics. Responses of TPP(H)A^−^/RPR^−^, TPP(H)A^+^/RPR^−^, and TPP(H)A^+^/RPR^+^ individuals to rp17 (left) and TmpA (right). Symbols represent data from a single individual. Individuals were grouped by reactivity to standard treponemal (TPPA or TPHA) or nontreponemal (RPR) diagnostics tests. Median fluorescent intensities against rp17 and TmpA treponemal antigens were plotted. Dotted lines denote cutoffs for positive responses. *n* = 586; 6 specimens testing TPP(H)A^−^/RPR^+^ were not included.

Specimens were stratified based on their combined reactivity in TPP(H)A and RPR assays and compared to responses obtained against rp17 and TmpA in the MBA assays. Among specimens testing dual negative by TPP(H)A and RPR, 97.6% were nonreactive against rp17, and 99.2% were nonreactive against TmpA (Fig. 1). The majority of TPP(H)A/RPR dual-positive specimens were reactive against rp17 (88.5%) and TmpA (70.6%). For TPP(H)A-positive/RPR-negative specimens, 75.0% had rp17-specific responses, whereas only 30.7% had TmpA-specific responses. The median MFI-BG for TmpA responses was lower in the TPP(H)A-positive/RPR-negative specimens (MFI-BG = 101) than in the dual-positive specimens (MFI-BG = 4,315).

Carboxylated cardiolipin was coupled to carboxylated microspheres using two different diamine linkers: poly-l-lysine and cadavarine. Cardiolipin-coated beads were run with control sera from known RPR-positive and RPR-negative individuals (see Fig. S1 in the supplemental material). When a cadavarine diamine linker was used, the MFI-BG of IgM responses to cardiolipin in RPR-positive serum decreased with increasing serum dilutions (1:6, 1:25, and 1:100). Anticardiolipin responses in the RPR-negative group were not detectable at the 1:400 dilution (see Fig. S1). Similar results were observed when poly-l-lysine was used as the diamine linker (data not shown). Anticardiolipin IgG responses were not detectable at any serum dilution (see Fig. S1).

### Comparison of treponemal antibody responses to RPR titers.

**FIG 2:**
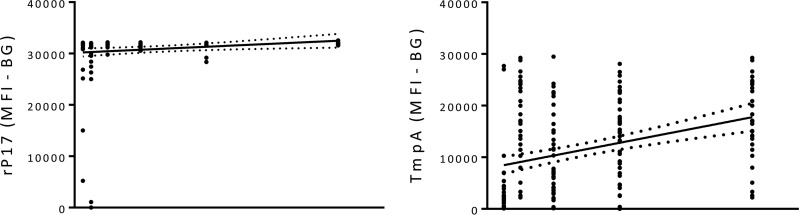
Linear regression analysis of anti-treponemal antibody levels against RPR titers. RPR titers are plotted against antibody responses against rp17 (left) or TmpA (right). Only data from TPP(H)A^+^ individuals were used in the analysis. Dots represent data from a single individual. The *x* axes are labeled with RPR titers, and *y* axes show MFI-BG of anti-treponemal antibody responses. Dotted lines represent 95% confidence intervals. Slopes of the two curves are significantly different (*P* < 0.0034). Number of specimens for each group: 88 RPR negative, 24 R1, 8 R2, 24 R4, 41 R8, 32 R16, 41 R32, 46 R64, and 30 R128.

Specimens were stratified by RPR titer and then compared to the intensity of MFI-BG of rp17 and TmpA responses. Linear regression analysis showed a positive correlation (*R*^2^ = 0.41; *P* < 0.0001) between TmpA MFI-BG levels and RPR titers (Fig. 2). A positive correlation between rp17 MFI-BG levels and RPR titers was also observed (*R*^2^ = 0.10; *P* < 0.0001) (Fig. 2). The slope of the regression line for TmpA (131.0 ± 8.5) was significantly steeper than that for rp17 (84.8 ± 12.7; *P* = 0.0034).

## DISCUSSION

In communities from three different countries in which yaws is endemic, antibody responses against the treponemal recombinant antigens rp17 and TmpA were detected with high sensitivity and specificity compared to responses with standard reference tests. Specimens with negative RPR titers typically had low-intensity values for TmpA, which may reflect a loss of TmpA-specific antibodies over time. In addition, we found a positive correlation between increasing RPR titers and increasing TmpA levels on an MBA assay, supporting that this treponemal antibody response might increase or decline in concert with RPR. Our data show that an MBA assay using two treponemal antigens, rp17 as a marker of historical infection and TmpA as a marker of recent infection, has the potential to be used as a single high-throughput screening tool for yaws programs.

Previous studies showed a decrease in TmpA titers in syphilis patients 3 to 9 months after treatment (11) and that specimens that were nonreactive in the nontreponemal VDRL test had lower TmpA levels than seen in VDRL-reactive specimens (12). The current report provides further evidence that antibody responses against TmpA are associated with low RPR titers and therefore may potentially be used to differentiate active or recent infections from past infection.

Surprisingly, there was also a lower intensity of rp17 MFI-BG in the TPP(H)A-positive/RPR-negative and R1- and R2-positive populations than with higher RPR titers. It is possible that even the traditional long-lived anti-treponemal antibody response wanes to some extent after resolution of infection but that this declining response is detectable only on highly sensitive assays, such as the MBA used in this study. The correlation between RPR titers and TmpA MFI-BG was much stronger than for rp17 MFI-BG, as the differences in the slopes of the regression lines were significant. While anti-TmpA responses approached seroreversion, anti-rp17 responses remained at high levels even at the 1:400 dilution used in this assay.

The ability to use TmpA to track recent or active infection would be valuable to programs, since this antigen can be incorporated into a high-throughput multiplex assay that can simultaneously measure responses to multiple analytes (i.e., to screen for several infections) in a single run. Therefore, a change in diagnostic procedure from the currently implemented RPR test that measures one specimen at a time to an MBA using TmpA has clear advantages. The BioPlex 2200 system, an automated multiplex instrument, was previously evaluated for automated syphilis diagnostics using commercially available treponemal antigen-coated microbeads to detect IgG for rp17 and other treponemal antigens and IgM against cardiolipin (13, 14). However, using this system for large-scale screening during a worldwide yaws eradication campaign presents several problems. First, this system still requires separate runs for the treponemal and nontreponemal antigens due to different secondary antibody requirements (detection of IgG versus IgM) and different dilutions needed to detect IgG versus IgM. In contrast, the ability to use anti-TmpA IgG testing for recent or active infection allows this test to be integrated with the treponemal antigen testing for yaws. Additionally, the cost per unit for the BioPlex 2200 and the corresponding commercially available reagents is high. In-house antigen-bead couplings are cost-effective and allow for use with a more affordable and adaptable multiplex-capable instrument, such as the Luminex or MagPix system. The methodology described herein would further allow the integration of yaws surveillance with other disease surveillance programs or vaccine coverage surveys, which use serology testing and often also focus on young children.

One limitation of this study was the relatively small number of specimens tested with the lowest RPR titers (especially R2, for which *n* = 8), which indicated very low or waning anticardiolipin levels. Having more of these specimens in future studies will allow us to get better estimates of TmpA antibody levels in this range, which in turn will increase the confidence that TmpA responses wane along with RPR titers. Another limitation is that we did not follow up with individuals after treatment over time, which, in our view, is the most accurate way to assess the dynamics of serology tests before and after treatment. Studies are being planned to evaluate the change in TmpA titers after treatment in individuals.

The renewal of the yaws eradication program has catalyzed advances in diagnostic tools for treponemal infections. Current serological diagnostics fulfill some but not necessarily all program needs. A combined rapid test, the dual-path platform syphilis assay, which detects both treponemal and nontreponemal antibodies, has been evaluated for the diagnosis of yaws and has shown high accuracy to confirm yaws cases in the field (15, 16). This type of field-deployable test will be critical for village-level evaluations during intensive programs monitoring both clinical cases and serologically positive cases of yaws over the course of 3 years where the program has been implemented (6). In contrast, a large-scale screening tool, such as that presented here, may benefit yaws eradication programs as a gross mapping tool to help determine where to conduct intensive screenings, since implementing intensive village-level surveillance in all countries in which yaws was previously endemic, current status unknown, is impractical. In addition, such a tool that can differentiate previous from current infection, if applied in appropriate age groups (children aged <5 years), might be used to assess ongoing transmission. WHO criteria to certify interruption of transmission include no RPR seroreactors among children aged <5 years, given that seroreactivity in young children can indicate recent infection as they are new entrants in the potential pool of susceptible individuals. In summary, this multiplex-based antibody testing may be useful during the first stage of the program to direct mapping efforts and during the endgame to certify the elimination of yaws.

## Supplementary Material

Supplemental material
