# Supplementary material for: Evaluation of Multiplex-Based Antibody Testing for Use in Large-Scale Surveillance for Yaws: a Comparative Study
Source: J Clin Microbiol. 2016 Apr 25;54(5):1321–5. doi: 10.1128/JCM.02572-15 (PMC4844712; doi:10.1128/JCM.02572-15)
Supplement: Supplemental material [file supp_54_5_1321__index.html]

Supplemental material 

# Evaluation of Multiplex-Based Antibody Testing for Use in Large-Scale Surveillance for Yaws: a Comparative Study

## Supplemental material

- Supplemental file 1 -

  Fig. S1 (Response of individuals who were negative or positive for RPR to cadaverine aminated beads bound to carboxyl cardiolipin)

  PDF, 199K
