# Supplementary material for: Evaluation of Multiplex-Based Antibody Testing for Use in Large-Scale Surveillance for Yaws: a Comparative Study
Source: J Clin Microbiol. 2016 Apr 25;54(5):1321–5. doi: 10.1128/JCM.02572-15 (PMC4844712; doi:10.1128/JCM.02572-15)
Supplement: Supplemental material [file JCM.02572-15_zjm999094945so1.pdf]

Supplemental Figure 1

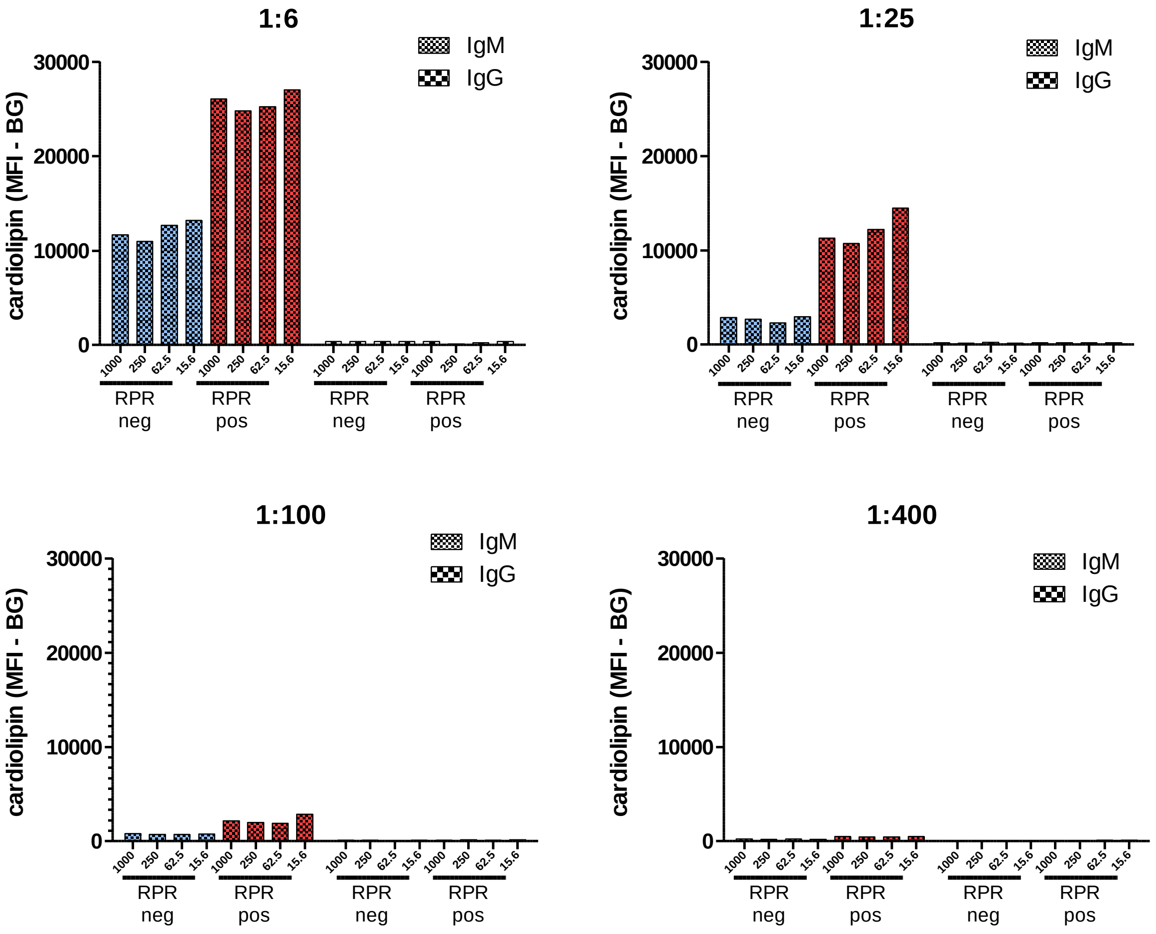

Supp. Figure 1. Response of individuals who were negative (blue bars) or positive (red bars) for RPR to cadaverine aminated beads bound to carboxyl cardiolipin. Sera from a normal RPR negative individual (blue bars) and sera from a syphilis positive, RPR positive individual (red bars) were run at four different dilutions with cardiolipin coated beads looking for IgM and IgG responses. Poly-L-lysine aminated beads (not shown) had similar results. Results shown are representative of N=12 RPR-positive specimens tested.
